# Diagnosis of Familial Hypercholesterolemia in Children: From Clinical Features Through Gene Variants to Polygenic Score

**DOI:** 10.3390/genes17030267

**Published:** 2026-02-26

**Authors:** Raffaele Buganza, Cecilia Nobili, Giulia Massini, Giovanna Cardiero, Maria Donata Di Taranto, Luisa de Sanctis, Ornella Guardamagna

**Affiliations:** 1 Department of Public Health and Pediatric Sciences, University of Turin, Regina Margherita Children’s Hospital, 10126 Turin, Italy; raffaele.buganza@unito.it (R.B.); cecilia.nobili@unito.it (C.N.); giulia.massini@unito.it (G.M.); luisadesanctis@unito.it (L.d.S.); 2 Dipartimento di Medicina Molecolare e Biotecnologie Mediche, Università degli Studi di Napoli Federico II, 80131 Naples, Italy; cardiero@ceinge.unina.it (G.C.); mariadonata.ditaranto@unina.it (M.D.D.T.); 3 CEINGE-Biotecnologie Avanzate Franco Salvatore, 80145 Naples, Italy

**Keywords:** familial hypercholesterolemia, children, gene variants, polygenic score, BMI

## Abstract

Background: Early diagnosis of familial hypercholesterolemia (FH) is crucial to improve long-term outcomes. FH diagnosis relies on elevated low-density lipoprotein cholesterol (LDL-C) levels, familial clinical characteristics, and identification of pathogenic variants in FH-related genes. Secondary factors, such as overweight and obesity, are known to influence lipid profiles in the general population. More recently, polygenic risk scores based on single-nucleotide polymorphisms (SNPs) have been proposed as additional determinants of LDL-C levels. Methods: We enrolled 214 pediatric subjects with LDL-C levels ≥95th percentile (after 6 months of dietary intervention) and with at least one parent with LDL-C levels ≥ 95th percentile. All participants underwent biochemical and auxological assessment and genetic testing for FH. In a subgroup of 60 subjects, LDL-C polygenic scores based on 6- and 12-SNPs were calculated. Results: Pathogenic variants confirming heterozygous FH were identified in 190 subjects (variant-positive, V+); 17 were variant-negative (V−), yielding a mutation detection rate of 91.8%. An additional seven patients carrying variants of uncertain significance were excluded from the primary analysis. LDL-C was modestly higher in V+ than V− subjects using both Friedewald (212 vs. 188 mg/dL; *p* = 0.035) and Martin–Hopkins formulas (208 vs. 187 mg/dL; *p* = 0.041), while the other main clinical and laboratory parameters were similar. In V+, LDL-C was higher in subjects with null variants, compared to those with defective variants. Body mass index (BMI SDS) was inversely correlated with HDL-C (*p* < 0.001), and obesity (BMI z-score > 2 SDS) was associated with lower HDL-C and higher LDL-C, non-HDL-C, and ApoB. With regard to the polygenic scores, 12- and 6-SNP scores showed overlap between V+ and V−, and published cut-offs did not discriminate lipid severity in our population; however, in V+ subjects, the 12-SNP score acted as a phenotype modifier, being independently associated with higher LDL-C and non-HDL-C levels after adjustment for age, sex, and BMI SDS. Conclusions: In children selected by LDL-C ≥ 95th percentile, together with autosomal dominant familial hypercholesterolemia, genetic confirmation of FH is achieved in the vast majority of cases. Variant type (null vs. defective), BMI, and polygenic background contribute to phenotypic heterogeneity, supporting the need to address other factors alongside genetic diagnosis. Further validation is needed before polygenic scores can be implemented in routine clinical practice.

## 1. Introduction

Heterozygous familial hypercholesterolemia (HeFH) is characterized by elevated low-density lipoprotein cholesterol (LDL-C) levels [1] and has an estimated prevalence in the general population of approximately 1 in 200–300 individuals [1,2].

Given the strong association between both the magnitude and the duration of vascular exposure to LDL-C and the risk of atherosclerotic disease [3], together with evidence demonstrating that atherosclerotic changes begin during childhood in individuals affected by HeFH [4], early diagnosis in childhood is crucial to enable the timely initiation of treatment and thereby reduce long-term cardiovascular risk.

Despite this evidence, HeFH remains severely underdiagnosed and undertreated [5]. Childhood represents the optimal period to discriminate between HeFH and non-HeFH individuals on the basis of LDL-C concentrations, owing to minimal environmental and hormonal influences; however, significant challenges persist, including the lack of standardized screening strategies and universally accepted clinical criteria for diagnosis and for selecting candidates for molecular analysis [6], which remains the gold standard for the diagnosis of FH [1].

The genes involved in FH include *LDLR* (which accounts for more than 90% of FH cases), *APOB* (responsible for approximately 5% of cases), and *PCSK9* (which contributes to less than 1% of cases), all inherited in an autosomal dominant manner, in addition to the extremely rare *LDLRAP1* variants, which exhibit an autosomal recessive pattern of inheritance [1].

Across different cohorts, approximately 5–30% of individuals with a phenotypic presentation consistent with HeFH do not harbor pathogenic variants in known FH-associated genes [1]. These cases may be explained either by variants in genes that have not yet been identified or by a polygenic etiology [1]. The cumulative contribution of multiple common variants with small individual effects, referred to as single-nucleotide polymorphisms (SNPs), can lead to increased LDL-C levels [7]. Based on this concept, several weighted polygenic risk scores (PRSs) have been developed to estimate the overall genetic predisposition to elevated LDL-C. Although findings are not entirely consistent and show variability across populations (possibly reflecting differences in genetic background among geographic regions), available data indicate that PRSs can account for hypercholesterolemia in a subset of genetically negative FH patients and partially explain the variability in LDL-C levels observed in individuals with monogenic HeFH [8,9,10].

Additional factors influencing lipid profiles in the general population include lifestyle-related variables; notably, increased body mass index (BMI) is associated with higher LDL-C, total cholesterol (TC), triglycerides (TGs), and non-HDL cholesterol (non-HDL-C), as well as lower HDL cholesterol (HDL-C), even during childhood [11]. Similar associations have more recently been described in FH patients, as reported by the European Atherosclerosis Society (EAS) FH Studies Collaboration Registry [12].

In this study, we further investigate the characteristics of individuals with suspected HeFH, identified using simple diagnostic criteria and referred for genetic testing for FH, by assessing the impact of genetic variants, PRS, and anthropometric parameters on lipid profiles.

## 2. Materials and Methods

### 2.1. Study Design

This was a retrospective observational study conducted in a pediatric population with suspected HeFH.

The study aimed to evaluate the impact of pathogenic variants, PRSs, and anthropometric parameters on lipid profiles.

Clinical, anamnestic, biochemical, and genetic data were collected from patients referred for FH genetic testing according to predefined diagnostic criteria, allowing for an integrated evaluation of genetic and non-genetic determinants of the HeFH phenotype.

All these data were collected as part of routine medical care and then retrospectively analyzed for the purposes of this study. The data were available in the clinical visit reports stored in the hospital’s information system.

**Figure 1:**
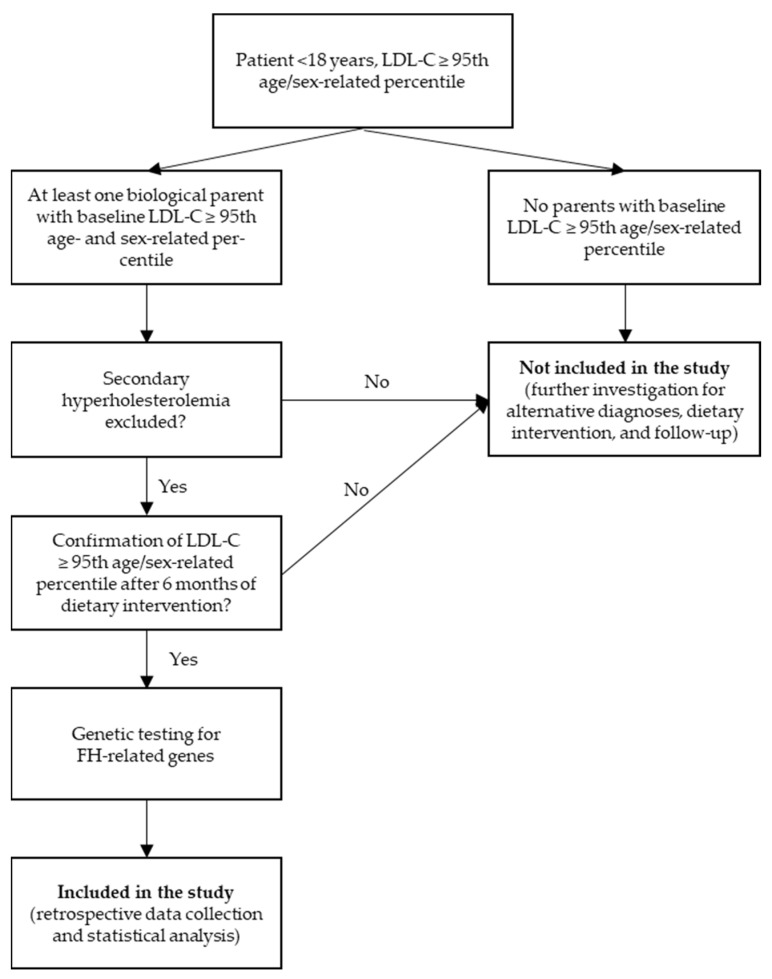
Flow chart of the study.

The study design is schematically illustrated in Figure 1.

### 2.2. Participants

The study included 214 pediatric subjects (age < 18 years) evaluated between 2004 and 2025 at the Lipid Clinic of the Regina Margherita Children’s Hospital (Turin, Italy), with a clinical suspicion of HeFH based on the following inclusion criteria, after 6 months of dietary intervention:

- LDL-C ≥ 95th age- and sex-related percentile [13];
- Autosomal dominant inheritance pattern of hypercholesterolemia, with at least one biological parent having baseline LDL-C ≥ 95th age- and sex-related percentile [14] (considering pre-treatment values in cases receiving lipid-lowering therapy).

In particular, the chosen percentiles for pediatric age are those reported by the Committee on Nutrition of the American Academy of Pediatrics (adapted from the Lipid Research Clinic Pediatric Prevalence Study) and routinely applied in our clinical practice, with 95th percentiles of LDL-C ranging from 129 to 140 mg/dL, depending on age and sex [13].

The dietary intervention was based on a Mediterranean-style diet and performed by an expert nutritionist in a face-to-face meeting during the first visit in our outpatient clinic. The recommended dietary pattern was characterized by a high intake of cereals (3–4 times/day); legumes (2–3 times/week); fish (2–3 times/week); plant foods (vegetables and fruit 4 times/day); nuts (20–30 g/day); extra virgin olive oil as the main source of fat (3–4 spoon /day); moderate consumption of fresh dairy products (2 times/day) and eggs (2–3/week); and a small amount of meat, especially red meat (1/week). Participants were instructed to limit sweets (allowed once per week); to avoid drinks with added sugar; and to exclude processed food, snacks, or junk food. 

Secondary hypercholesterolemia was ruled out clinically and through a panel of biochemical analyses to exclude renal and liver disease, hypothyroidism, and diabetes: creatinine, aspartate aminotransferase (AST), alanine aminotransferase (ALT), thyroid-stimulating hormone (TSH), free thyroxine (FT4), fasting plasma glucose, and glycated hemoglobin (HbA1c).

Combined dyslipidemia was excluded by including only patients with triglyceride levels within the normal range for pediatric age [13].

PRS evaluation was performed in a subgroup of 60 subjects. PRS genotyping was carried out at a single reference laboratory (CEINGE, Naples) and has been available since 2013, when NGS-based screening with concurrent SNP genotyping was implemented. Initially performed primarily for research purposes, PRS assessment was subsequently incorporated into the routine diagnostic genetic workup. PRS data were, therefore, unavailable for patients whose genetic analysis had been conducted before this period.

### 2.3. Clinical and Anamnestic Evaluation

We collected detailed medical histories from all patients, carefully documenting the presence of dyslipidemia and cardiovascular diseases in family members across two generations, distinguishing between premature and non-premature events, and obtaining parental lipid profiles when not previously available.

Personal medical history included assessment of other conditions that could represent secondary causes of dyslipidemia, current medications, dietary habits, and physical activity.

A comprehensive physical examination was then performed, including the assessment of specific clinical signs such as xanthelasmas, xanthomas, and corneal arcus, alongside anthropometric measurements.

Body weight was measured using a mechanical scale with a precision of 0.1 kg, in a standing position. Height was measured with a Harpenden stadiometer, with patients standing upright, and head positioned according to the Frankfurt plane. BMI was calculated as weight in kilograms divided by the square of height in meters (kg/m^2^). Anthropometric measures were compared to the World Health Organization (WHO) reference standards [15].

### 2.4. Biochemical Analysis

Blood samples were collected after an overnight fast. Lipid profile assessment comprised the measurement of TC, HDL-C, TG, ApoB, and lipoprotein (a)—Lp(a).

Plasma LDL-C concentrations (mg/dL) were calculated using both the Martin–Hopkins formula [LDL-C = TC − HDL-C − (TG/adjustable factor)] and the Friedewald formula [LDL-C = TC − HDL-C − (TG/5)], as will be highlighted in the Results section.

Non-HDL cholesterol levels were calculated by subtracting HDL-C from TC.

LDL-C values for patients and their parents were compared with age- and sex-specific population percentiles for children [13] and adults [14].

### 2.5. Genetic Testing

For patients in whom the genetic investigation was performed before 2019, in accordance with the routine medical care of our center for suspected HeFH, genetic screening was performed using a stepwise protocol based on traditional methods, as previously described [16]. Initially, the *LDLR* gene was analyzed by amplification and direct sequencing of all exons and adjacent intronic regions; if no pathogenic variants were detected, Multiplex Ligation-dependent Probe Amplification (MLPA) was performed to identify large rearrangements in the *LDLR* gene [16]. When *LDLR* variants were absent, sequencing of all exons and flanking intronic regions of the *PCSK9* gene was subsequently conducted [17]. If negative, *APOB* gene analysis was performed, including the region encoding the LDLR binding domain (portion of exon 26, exon 29, and flanking exon–intron junctions) [16].

For patients in whom the genetic investigation was performed from 2019 onwards, a next-generation sequencing (NGS)-based approach was used, employing the Devyser FH v2 kit for sequence enrichment and the Illumina MiSeq platform with paired-end reads (2 × 150 bp); this method allowed for the detection of small variants in the *LDLR*, *APOB*, *PCSK9*, *LDLRAP1*, *APOE*, and *STAP1* genes, as well as copy number variants in *LDLR* [6]. Sequence analysis was performed using the Amplicon Suite software, version 3.5.1 (SmartSeq s.r.l., Alessandria, Italy).

Rare variants were evaluated for pathogenicity according to FH-specific recommendations of the American College of Medical Genetics and Genomics (ACMG) via ClinGen [17,18]. Variant data were collected using the Genome Aggregation Database (GnomAD) for minor allele frequency (MAF) and by consulting pathogenic variant databases such as HGMD Professional, Leiden Open Variation Database (LOVD 3.0), and ClinVar for previously reported FH-associated variants [19].

The Devyser FH v2 kit was also used to genotype the following SNPs for the calculation of the LDL-C PRS based on previously published 12- and 6-SNPs:

- rs2479409 (PCSK9), rs629301 (*CELSR2*), rs1367117 (*APOB*), rs4299376 (*ABCG8*), rs1564348 (*SLC22A1*), rs1800562 (*HFE*), rs3757354 (*MYLIP*), rs11220462 (*ST3GAL4*), rs8017377 (*NYNRIN*), rs6511720 (*LDLR*), rs429358 (*APOE*), rs7412 (*APOE*);
- rs629301 (*CELSR2*), rs1367117 (*APOB*), rs6544713 (proxy of rs4299376, *ABCG5/8*), rs6511720 (*LDLR*), rs429358 (*APOE*), rs7412 (*APOE*).

After genotype retrieval using the Amplicon Suite software (SmartSeq s.r.l.), calculation of the two PRSs was performed using an Excel spreadsheet to multiply the number of risk alleles of each SNP by its corresponding weight, according to Talmud et al. [8] and Futema et al. [20].

To define PRS positivity or negativity, we considered the cut-offs for the Italian population derived from the studies by Olmastroni et al. [9] and Cardiero et al. [10].

### 2.6. Statistical Analysis

Continuous variables were described as mean ± standard deviation (SD) when approximately normally distributed, or as median with interquartile range (IQR) when non-normally distributed. Categorical variables were reported as counts and percentages.

Normality of continuous variables was assessed using the Shapiro–Wilk test. Comparisons between-group (e.g., pathogenic variant-positive vs. pathogenic variant-negative) were performed using the Mann–Whitney U tests for non-normally distributed variables. Categorical variables were compared using Pearson’s chi-square test.

Associations between continuous variables (e.g., PRSs and lipid parameters) were evaluated using Spearman’s rank correlation coefficient (ρ) with two-sided *p*-values.

To assess whether the 12-SNP polygenic risk score acted as a phenotype modifier among genetically confirmed cases, we performed multivariable linear regression analyses restricted to pathogenic variant-positive subjects with available data (complete-case approach per model). Separate models were fitted for LDL-C and non-HDL-C (mg/dL) as outcomes, with PRS entered as a continuous predictor and adjusting for age, sex, and BMI SDS.

Missing data were handled using complete-case analysis for each test/model, and the effective sample size used in each analysis was reported.

PRS data were available only for patients recruited after 2013, when NGS-based screening with SNP genotyping was implemented; the missingness mechanism is, therefore, primarily related to recruitment timing rather than clinical characteristics.

All tests were two-sided, with *p* < 0.05 considered indicative of statistical significance.

Given the exploratory nature of the study, no correction for multiple comparisons was applied; accordingly, findings should be interpreted with caution and confirmed in larger independent cohorts.

All analyses were performed using jamovi (The jamovi project), version 2.7.15.

## 3. Results

### 3.1. Characteristics of Patients and Genetic Testing Results

A total of 214 patients underwent molecular analysis according to the predefined inclusion criteria. Of these, 190 carried a causative variant (V+), 17 were tested negatives (V−), and 7 harbored a variant of uncertain significance (VUS).

The mutation detection rate (MDR) was calculated to be 91.8%.

Clinical characteristics and lipid profiles of the patients are summarized in Table 1.

For the main analyses and reported results, patients classified as VUS were excluded.

Sensitivity analyses were nevertheless performed to assess the robustness of findings under alternative VUS classifications (as V+ or V−). The seven VUS subjects showed LDL-C levels similar to V− subjects (*p* = 0.799) and lower levels than V+ subjects (*p* = 0.079), but sensitivity analyses confirmed that the primary findings remained robust regardless of VUS classification.

Among the 190 V+ cases, 187 harbored variants in the *LDLR* gene, and 3 in the *APOB* gene. Missense variants represented 55% of *LDLR* variants and 100% of *APOB* variants. Among *LDLR* variants, 63% were categorized as “defective” and 37% as “null”. The most frequent variants (identified in ≥5 subjects) included five “defective” *LDLR* variants: c.1567G>A (p.Val523Met) (FH Bari-2) in 20 patients, c.1646G>A (p.Gly549Asp) (FH Palermo-1) in 10 patients, c.1775G>A (p.Gly592Glu) (FH Sicily or Foggia-1) in 10 patients, c.1735G>T (p.Asp579Tyr) (FH Casale Monferrato) in 7 patients, and c.662A>G (p.Asp221Gly) (FH Padova-1) in 5 patients.

When comparing variant-positive (V+) and variant-negative (V−) subjects, the only laboratory parameter that showed a statistically significant difference was LDL-C, which was modestly higher in V+ subjects, 208 vs. 187 mg/dL (*p* = 0.041) with the Martin–Hopkins formula and 212 vs. 188 mg/dL (*p* = 0.038) with the Friedewald formula. LDL-C values calculated by the Friedewald and Martin–Hopkins formulas showed near-perfect correlation (r = 0.997), with the Friedewald formula yielding values 3.5 mg/dL higher on average.

No other statistically significant differences were detected in the main clinical or laboratory parameters.

Lp(a) concentrations were available for 76 V+ individuals, with a median of 26.6 (11.8–66.3) mg/dL. Of these, 25 (32.9%) had values >50 mg/dL, and 11 (14.5%) fell within the range 30–50 mg/dL. No significant difference in Lp(a) values was found between pediatric V+ subjects with and without a family history of CHD (*p* = 0.609) or pCHD (*p* = 0.421).

A high frequency of CHD and pCHD in parents and relatives was observed in both V+ and V− groups, as shown in Table 2, with no significant differences between the two groups. None of the pediatric participants experienced any cardiovascular events.

### 3.2. Evaluation of Further Factors Influencing Lipid Profile

Within the V+ group, carriers of “null” variants (including nonsense, splicing, and deletion/insertion leading to frameshift and large rearrangements) displayed significantly higher LDL-C concentrations than those with “defective” variants (including missense, deletion/insertion without frameshift, and promoter variants), with median values of 223 (190–264) vs. 201 (187–226) mg/dL (*p* = 0.003) according to the Martin–Hopkins formula and 226 (194–267) vs. 205 (189–230) mg/dL (*p* = 0.003) according to the Friedewald formula. No other relevant differences were identified across the remaining lipid measures between “null” and “defective” variants.

In V+ patients, Spearman correlation analysis between anthropometric indices and lipid profile showed a negative association between BMI SDS and HDL-C levels (*p* < 0.001).

Furthermore, V+ patients with a BMI z-score >2 SDS (n = 26), compared to those with a BMI z-score <2 SDS (n = 164), exhibited lower HDL-C levels, 49 (45–54) vs. 56 (48–65) mg/dL (*p* < 0.01), higher LDL-C levels, using both the Martin–Hopkins formula [226 (201–272) vs. 206 (187–235) mg/dL (*p* = 0.016)] and the Friedewald formula [227 (203–275) vs. 210 (190–239) mg/dL (*p* = 0.022)], higher non-HDL-C, 245 (217–295) vs. 224 (202–257) mg/dL (*p* = 0.019), and higher ApoB, 157 (144–175) vs. 140 (122–155) mg/dL (*p* = 0.042). No differences in TG or Lp(a) levels were detected between groups.

Regarding PRSs, the values in V+ and V− were as follows (expressed as medians with inter-quartile ranges):

- V+: 0.968 (0.834–1.093) for 12-SNP PRS and 0.681 (0.510–0.831) for 6-SNP PRS.
- V−: 0.967 (0.841–1.052) for 12-SNP PRS and 0.695 (0.563–0.730) for 6-SNP PRS.

No statistically significant differences were observed between V+ and V− for either the 12-SNP or 6-SNP PRS.

The 12-SNP PRS was positive in 32/52 (61.5%) V+ and 5/8 (62.5%) V− using both the 0.905 cut-off of Olmastroni et al. [9] and the 0.89 cut-off of Cardiero et al. [10].

When the 6-SNP PRS cut-off of 0.62 derived from Cardiero et al. [10] was applied, three patients changed classification: two V+ became positive, and another V+ became negative, while V− did not change PRS classification (in two subjects, only 12-SNP scores were available).

This 6-SNP PRS was positive in 33/52 V+ (63.5%) and 4/6 V− (66.7%).

In V+, while dichotomized cut-offs did not discriminate lipid severity, the continuous 12-SNP score showed a positive trend with lipid severity in univariate analyses (Spearman ρ = 0.26 with non-HDL-C, *p* = 0.063; ρ = 0.23 with LDL-C calculated by Friedewald, *p* = 0.107). Notably, this association reached statistical significance for LDL-C when it was calculated using the Martin–Hopkins formula (ρ = 0.31, *p* = 0.028). In multivariable linear regression restricted to V+ subjects and adjusted for age, sex, and BMI SDS, a higher 12-SNP score was independently associated with higher non-HDL-C (β = 62.6 mg/dL per 1-unit increase; equivalently, +6.3 mg/dL per +0.1; 95% CI +7.0 to +118.1; *p* = 0.028) with the Friedewald formula. The association with LDL-C calculated by Friedewald showed a consistent trend (β = 51.4 mg/dL per 1-unit; 95% CI −1.9 to +104.6; *p* = 0.058) and reached statistical significance when using the Martin-Hopkins formula (β = 72.5 mg/dL, 95% CI +16.8 to +128.1; *p* = 0.012). Within the subgroup with PRS values, only eight V− values were present, a sample too small for meaningful analysis.

## 4. Discussion

Given the increased cardiovascular risk associated with prolonged exposure to elevated LDL-C levels from childhood, it is crucial to identify the most effective approaches for diagnosing HeFH and selecting patients for molecular analysis, which provides the definitive diagnosis.

In the pediatric population, the combination of LDL-C levels ≥ 95th percentile in the proband and a dominant inheritance pattern of hypercholesterolemia represents a simple and effective diagnostic criterion, demonstrating high MDR (91.8%), comparable to more complex and time-consuming clinical criteria used in practice, which often involve higher LDL-C or TC cut-offs, potentially leading to missed diagnoses in some individuals [6].

Various studies report highly variable MDR for different criteria, with a greater proportion of patients testing negative observed among adult populations. However, Van der Graaf et al., in a study of 1430 children with LDL-C above the 95th percentile and an autosomal dominant inheritance pattern of hypercholesterolemia (at least one biological parent on treatment for hypercholesterolemia and a family history of hypercholesterolemia and cardiovascular disease), reported an MDR of 95%, higher than in previous studies, likely due to more stringent selection criteria and more comprehensive molecular genetic analyses [21]. These findings indicate that patients with moderately elevated LDL-C but clear autosomal dominant transmission of hypercholesterolemia should also be evaluated for genetically based HeFH. Wiegman et al. showed that LDL-C levels >3.50 mmol/L (~135 mg/dL) had a 98% post-test probability of detecting the presence of an *LDLR* pathogenic variant in children from families with known HeFH [22]. However, in routine practice, genetic confirmation in parents is often unavailable, making it necessary to rely primarily on the lipid profile.

It should be noted that many diagnostic criteria use higher LDL-C cut-offs, while the 95th percentile LDL-C threshold allows for the identification of more individuals while maintaining excellent diagnostic performance in pediatric age [6]. In the future, it could be considered whether further lowering the cut-off in children and adults might help capture even more individuals carrying FH variants, given that the presence of FH variants is associated with higher CHD risk in adults, even when LDL-C is only moderately elevated [23]. In such cases, genetic testing may refine risk stratification and, with early treatment, may subsequently reduce excess CHD-related mortality [23]. This approach could reduce diagnostic specificity and MDR, but the previous cost burden of genetic testing is decreasing, and future assessments may allow for greater access and ease of testing, particularly in pediatric FH, where early identification is crucial.

Regarding causal genes, *LDLR* was the most frequently involved in our study (98.5%), consistent with previous reports from LIPIGEN studies in Italy, where over 94% of detected pathogenic variants affected *LDLR* [24]. The most common type of variant was missense (55%), in line with the literature data (a previous Italian study reported 59.9% [25]).

Comparing *LDLR* “null” versus “defective” variants, patients with “null” variants exhibited significantly higher LDL-C levels than those with “defective” ones, consistent with the prior literature, which showed that carriers of *LDLR* “null” variants had a more severe phenotype, in terms of plasma lipid levels and intima-media thickness (IMT), and a higher prevalence of pCHD in first-degree relatives, warranting closer clinical monitoring and early pharmacologic intervention [26].

In our cohort, LDL-C values were higher in V+ than V−, with no other significant differences between the two groups. The prevalence of familial CHD was slightly higher in the V− group compared to the V+ group, but the difference was not statistically significant. This point is of relevance, as the absence of CHD should not reduce clinical suspicion of HeFH.

A novel diagnostic dimension is emerging through polygenic risk scores (PRSs) derived from SNPs. In line with evidence from various studies, higher scores should be regarded as predisposing factors for hypercholesterolemia in subjects without FH variants and may modulate the clinical phenotype even in patients with monogenic HeFH, highlighting that, beyond the classic genetic lipid abnormalities, additional factors can negatively influence the lipid profile [8,9,10]. These findings suggest the potential clinical relevance of PRS, though implementation remains limited due to overlapping distributions between V+ and V− patients and a lack of validated cut-offs for reliably distinguishing polygenic forms.

In our cohort, we applied the cut-offs derived from previous studies in the Italian population by Olmastroni et al. for 12-SNP PRS [9] and Cardiero et al. for 12-SNP and 6-SNP PRSs [10]. Using these thresholds, PRSs showed substantial overlap between V+ and V−, and published cut-offs did not discriminate lipid severity in our population. However, when treated as a continuous measure, the 12-SNP score behaved as a phenotypic modifier among V+ patients, being independently associated with non-HDL-C, and also with LDL-C, when calculated using the Martin–Hopkins formula.

The findings of our study support a combined monogenic–polygenic model, where the causal FH variant drives the LDL-C elevation, but polygenic background may modulate the atherogenic lipid burden and help to explain inter-individual variability among pathogenic variant carriers. In this context, our data suggest that the PRS should be better framed as a modifier of lipid severity rather than as a diagnostic discriminator.

In the current clinical practice, high PRS values do not influence treatment strategies in children with FH, which are aimed at LDL-C reduction through lifestyle interventions and lipid-lowering therapy. Additional considerations may be required in subjects classified as V− or in the presence of other cardiovascular risk factors. In these settings, PRSs may improve risk stratification and help tailor the clinical approach; however, further studies are warranted to clarify the impact of the PRS on cardiovascular risk.

Olmastroni et al. [9] and Cardiero et al. [10] also reported statistically significant correlations between PRSs and LDL-C levels.

Using the Friedewald equation, this association did not reach statistical significance in our cohort; however, when LDL-C was calculated using the Martin–Hopkins formula, the correlation with PRS became significant, suggesting that the choice of LDL-C estimation method may influence the detection of genotype-phenotype associations.

Residual differences compared with the studies of Olmastroni et al. [9] and Cardiero et al. [10] may be attributable to the smaller sample size of our cohort, as well as to methodological and population differences. Olmastroni et al. [9] included mainly adults, whose lipid profile is more likely to be influenced by lifestyle and age-related factors, effects that may be more pronounced in the presence of a predisposing polygenic background. Cardiero et al. [10] enrolled a broader “clinical suspicion” cohort that included individuals with lower LDL-C thresholds (LDL-C ≥ 120 mg/dL for children), while our cohort focused on children and parents with LDL-C ≥ 95th percentile, leading to a very high pre-test probability for monogenic FH. In such an enriched setting, the discriminatory performance of the PRS is expected to be attenuated due to range restriction, a small and selected gene-negative subgroup, and the dominant contribution of the causal variant to LDL-C levels among pathogenic-variant-positive subjects.

An additional methodological consideration emerging from this study concerns the estimation of LDL-C levels. The Friedewald formula, while widely used, assumes a fixed triglyceride-to-VLDL-C ratio of 5:1 that does not account for inter-individual variability [27]. In pediatric FH populations with normal triglyceride levels, this assumption typically leads to LDL-C overestimation; importantly, the magnitude of this error varies across individuals depending on their triglyceride concentration, potentially attenuating genotype–phenotype correlations. The Martin–Hopkins formula, which uses an adjustable factor based on triglyceride and non-HDL-C strata, has demonstrated superior accuracy across diverse populations, including pediatric cohorts and patients with elevated LDL-C levels [27]. In our study, PRS associations with LDL-C, which showed only a trend using the Friedewald formula, reached statistical significance with the Martin–Hopkins formula, both in univariate correlation (*p* = 0.107 vs. *p* = 0.028) and multivariable regression analysis (*p* = 0.058 vs. *p* = 0.012). Recent data suggest that discrepancies between Friedewald and Martin–Hopkins estimates may also carry diagnostic relevance in pediatric FH [28]. These observations suggest that the choice of LDL-C calculation method may be particularly relevant in FH populations, where accurate phenotyping is essential for both clinical management and research purposes.

Additional findings from this study include a negative correlation between BMI and HDL-C in HeFH patients, consistent with the known reduction in HDL-C with increasing body weight in the general population [11] and in HeFH subjects in the recent publication by the EAS Registry [12], while the correlations with other lipid parameters reported by the EAS Registry [12] were not detected in our study. In our cohort, obese HeFH patients showed lower HDL-C and higher LDL-C, non-HDL, and ApoB compared to non-obese subjects, reflecting lipid abnormalities associated with excess weight [11]. TG differences were not observed, likely because normal TG was an inclusion criterion.

Compared with data from the EAS Registry, we also investigated ApoB levels in HeFH subjects, finding higher values among obese individuals. While LDL-C reflects only a portion of atherogenic cholesterol, ApoB reflects the total atherogenic particle number, independent of the cholesterol content. Evidence also indicates that ApoB is a more accurate marker of all-cause mortality risk in statin-treated patients than LDL-C or non-HDL-C and a better predictor of myocardial infarction risk than LDL-C [29].

These results highlight that, beyond genetic lipid abnormalities, additional factors can negatively influence the lipid profile. Therefore, in HeFH patients, who already carry increased genetic risk, it is especially important to minimize additional risk factors, making nutritional counseling and physical activity essential. Diet is a cornerstone of the management of FH in children [1], and adherence to a heart-healthy diet in adults has been associated with lower concentrations of atherogenic lipids and lipoproteins, as well as a reduced risk of CHD among individuals with clinical HeFH, independently of lipid-lowering therapy [30].

Most evidence regarding the effects of diet on the lipid profile and cardiovascular risk comes from studies in adults [31]. The EAS Consensus for FH in children and adolescents recommends that dietary interventions emphasize a culturally acceptable heart-healthy diet, such as a Mediterranean-style pattern [1], and a previous investigation on the Mediterranean diet carried out at our institution in Turin reported improvements in lipid parameters in primary dyslipidemia, including FH, particularly associated with a lower intake of saturated fats [32]. These findings highlight the importance of reinforcing nutritional counseling as a key component of FH management, in synergy with pharmacological lipid-lowering therapy.

This study has some limitations. Its single-center design and the small sample size of variant-negative subjects (n = 17) limit statistical power and generalizability. Patients referred to a specialized pediatric lipid clinic may not be representative of the general HeFH population. PRS data were available only for patients whose genetic analysis was performed from 2013 onwards at a single reference laboratory. VUSs were excluded from primary analyses; sensitivity analyses confirmed the robustness of the findings, with VUS patients showing a phenotype similar to V− subjects. Overall, the findings should be considered exploratory pending confirmation in larger, multicenter cohorts.

## 5. Conclusions

Early detection of pediatric patients with HeFH is crucial for the timely initiation of treatment and for reducing long-term cardiovascular risk. In the pediatric population, the combination of LDL-C ≥ 95th percentile in the proband and a dominant inheritance pattern of hypercholesterolemia represents a simple and effective diagnostic criterion, demonstrating a high mutation detection rate (MDR).

In this enriched pediatric cohort, polygenic LDL-C scores showed limited diagnostic value in distinguishing variant-positive from variant-negative subjects using previously published cut-offs. Nonetheless, the polygenic background may still contribute to inter-individual variability in lipid severity among variant-positive patients, as shown by the independent association between PRS and both non-HDL-C and LDL-C levels. Further research is required to translate polygenic scores into clinical practice.

Beyond inherited risk, modifiable factors, especially excess body weight, were associated with a more atherogenic lipid profile. Comprehensive care in pediatric HeFH should, therefore, combine genetic diagnosis and LDL-lowering therapy with structured lifestyle interventions aimed at optimizing BMI and overall cardiovascular risk.

## Figures and Tables

**Table 1:** Characteristics of index patients.

	Overall	Pathogenetic Variant (V+)	No Variant (V−)	*p*-Value
Number	207	190	17	
Age (years)	10.5 (7.6–13.3)	10.4 (7.2–13.3)	11.5 (9.7–15.8)	0.186
Sex (male/female)	105/102	97/93	8/9	0.752
Height (cm)	145.0 (127.0–158.0)	143.5 (127.0–157.8)	147.0 (137.7–152.8)	0.738
Height (SDS)	0.2 (−0.6–0.8)	0.2 (−0.7–0.8)	0.3 (−0.0–0.8)	0.646
Weight (kg)	38.8 (27.1–52.6)	38.5 (26.6–52.7)	41.0 (36.0–49.6)	0.377
Weight (SDS)	0.4 (−0.3–1.3)	0.4 (−0.3–1.3)	1.2 (0.3–1.7)	0.119
BMI (kg/m^2^)	18.6 (16.0–21.1)	18.6 (16.0–21.1)	19.1 (17.9–21.5)	0.244
BMI (SDS)	0.4 (−0.4–1.3)	0.4 (−0.4–1.2)	1.2 (−0.2–1.8)	0.159
TC (mg/dL)	285 (257–314)	286 (257–315)	275 (251–296)	0.176
LDL-C (mg/dL)—MH	207 (187–241)	208 (188–243)	187 (173–221)	0.041 *
LDL-C (mg/dL)—F	211 (189–245)	212 (191–246)	188 (176–224)	0.038 *
HDL-C (mg/dL)	55 (48–64)	55 (48–64)	60 (47–77)	0.252
TG (mg/dL)	72 (57–91)	72 (56–90)	81 (60–112)	0.345
Non-HDL-C (mg/dL)	225 (203–261)	226 (203–261)	214 (192–243)	0.074
Lp(a) (mg/dL) °	28.1 (11.8–63.8)	26.6 (11.8–66.2)	41.8 (19.4–52.5)	0.831
Apo B (mg/dL) ^	142 (123–158)	144 (125–158)	131 (114–152)	0.459

MH: calculated with Martin–Hopkins formula; F: calculated with Friedewald formula; * *p*-value significant (<0.05); ° available in 76 V+ and 8 V−; ^ available in 89 V+ and 8 V−.

**Table 2:** Coronary Heart Disease (CHD) and precocious CHD (pCHD) in proband kindreds.

	Overall	V+	V−	*p*-Value
CHD in I–II-degree relatives (number, %)	141 (68.1%)	128 (67.3%)	13 (76.4%)	0.590
pCHD in I–II-degree relatives (number, %)	103 (49.8%)	94 (49.4%)	9 (52.9%)	0.806
pCHD in parents, (number, %)	33 (15.9%)	30 (15.7%)	3 (17.6%)	0.738

## Data Availability

The data are available from the corresponding author upon reasonable request.
